# What does delirium cost? An economic evaluation of hyperactive delirium

**DOI:** 10.1007/s00391-015-0871-6

**Published:** 2015-03-24

**Authors:** W. Weinrebe, E. Johannsdottir, M. Karaman, I. Füsgen

**Affiliations:** 1 Innere Medizin, Hohenloher Krankenhaus gGmbH, Krankenhaus Künzelsau, Universität Witten-Herdecke, Am Sonnenrain 28, 74909 Meckesheim, Deutschland; 2 Institut für Biomathematik Berlin, Berlin, Deutschland; 3 Geriatrische Medizin, Marien Hospital Bottrop, Universität Witten-Herdecke, Herdecke, Deutschland

**Keywords:** Delirium, Costs, Hospitalization period, Personnel expenses, Delir, Kosten, Krankenhausverweildauererweildauer, Personalaufwand

## Abstract

**Background:**

Demographic changes have resulted in an increase in the number of older (> 75 years) multimorbid patients in clinics. In addition to the primary acute diagnoses that lead to hospitalization, this group of patients often has cognitive dysfunctions, such as delirium. According to clinical experience, delirium patients are more time-consuming for clinicians and their function is often poor. The costs caused by delirium patients are currently unknown. In the present study, a retrospective examination of a database was carried out to calculate the costs that arise during the clinical treatment of documented delirium patients.

**Setting and methods:**

The purpose of this retrospective analysis was to collect information recorded by nursing personnel trained in the treatment of delirium and information from a manual documentation matrix for additional time expenditure. In the database analysis anonymous data of previously discharged patients for a time window of 3 months were analyzed. Documented additional expenditure for patients with hyperactive delirium at hospitalization were analyzed by personnel. Material costs, the duration of hospitalization by main diagnosis and age clusters during hospitalization until discharge were also examined. The analysis was performed in a hospital with internal wards.

**Results:**

Data for 82 hyperactive delirium patients were examined and an average of approximately 240 min of additional personnel expenditure for these patients was found. These patients were approximately 10 years older (*p* < 0.01) and were hospitalized for an average of 4.2 days longer (*p* < 0.01) than non-delirium patients. Hyperactive delirium usually developed within the first 5 days of hospitalization and lasted 1.6 days on average. Patients for whom hyperactive delirium was detected early were hospitalized for significantly less time than those for whom it was detected late (6.85 versus 13.61 days, *p* = 0.002). Additionally, calculated personnel and material costs, including costs affecting the hospitalization period, amounted to approximately 1200 € per hyperactive delirium patient. This corresponds to approximately 0.3 CMP (casemix points) per patient.

**Conclusion:**

The calculations of personnel and material costs and duration of hospitalization in patients with hyperactive delirium demonstrated significant additional costs. Early routine detection of delirium can be achieved through training and this approach leads to a shortening of the hospitalization period and lower costs.

## Abstract (de)

**Hintergrund:**

Die demografischen Veränderungen führen zu mehr hochbetagten (> 75 Jahre), multimorbiden Patienten in der Klinik. Diese Patientengruppe zeigt neben den akuten Einweisungshauptdiagnosen (HD) häufig kognitive Funktionsstörungen, z. B. ein Delir. Klinischer Erfahrung zufolge sind Delirpatienten aufwendiger, oft sind sie funktionell stark beeinträchtigt. Wie viel Delirpatienten an Kosten verursachen, ist bisher nicht bekannt. In der vorliegenden Arbeit wurde retrospektiv berechnet, welche Kosten bei der klinischen Versorgung dokumentierter Delirpatienten anfielen.

**Setting und Methoden:**

Grundlage für diese retrospektive Auswertung war speziell auf die Erfassung von Delirien geschultes Pflegepersonal und eine händische Dokumentationsmatrix für zusätzlichen Zeitaufwand. In der Datenbankanalyse wurden die anonymisierten Daten von bereits entlassenen Patienten einer allgemeinen Innere Abteilung in einem Krankenhaus der Grund- und Regelversorgung aus einem Zeitfenster von 3 Monaten ausgewertet. Bei Vorliegen von hypermotorischen Delirien in der Aufnahmesituation bzw. im klinischen Verlauf bis zur Entlassung wurden die dokumentierten Zusatzaufwände hinsichtlich Personal, Material und Verweildauer nach HD und Altersclustern ausgewertet.

**Ergebnisse:**

Untersucht wurden Daten von 82 hypermotorischen Delirpatienten, die durchschnittlich etwa 240 Minuten an zusätzlichem Personalaufwand benötigten. Diese Patienten waren gegenüber Nicht-Delirpatienten etwa 10 Jahre älter (*p* < 0,01) und lagen im Durchschnitt 4,2 Tage länger (*p* < 0,01) im Krankenhaus. Hypermotorische Delirien traten meist innerhalb der ersten 5 Tage auf und dauerten durchschnittlich 1,6 Tage; bei früh diagnostiziertem Delir signifikant kürzer als bei spät erkanntem (6,85 vs. 13,61 Tage; *p* = 0,002). Zusätzlich errechnete Personal- und Sachkosten beliefen sich einschließlich der Verweildauerbeeinflussung auf etwa EUR 1200,00 pro hypermotorischem Delirpatienten, entsprechend etwa 0,3 CMP pro Patient.

**Schlussfolgerung:**

Die Kalkulation der Bereiche Personal, Sachkosten und Verweildauer bei Patienten mit hypermotorischen Delirien zeigt die erheblichen Zusatzkosten bei Delirpatienten auf. Eine frühe, routinemäßige Erfassung von Delirien kann durch eine Schulung erfolgreich gelingen, führt zu spezifischen Prozessen, verkürzt die Verweildauer und senkt die Kosten.

## Background

The average age of clinically treated patients has increased regardless of the specific clinical activity (e.g. internal, surgical and urological) [6]; therefore, an increase in age-related comorbidities can be expected. Delirium is a significant, common [2, 12, 15, 18] and dangerous complication [1, 3, 11, 20] in acutely ill, older patients and is associated with significant additional expenses. It is not often considered [17, 19] and to date there are no exact investigations of the time-related additional expenses by the medical and nursing personnel with respect to the treatment of delirious patients in the German or international literature, with the exception of rough calculations [14, 16]. There are also no data on the exact use of materials that can be attributed to the treatment of delirium. The present study was an initial attempt to retrospectively collect information regarding additional expenses and costs directly associated with the treatment of hyperactive delirium to perform an economic evaluation of costs associated with this disease.

## Study design and method

This study was a retrospective database analysis of an internal medical ward, including an intensive care unit (ICU), stroke unit, geriatric medicine, with 2300 admissions/year and a mean length of stay of 7.2 days, on discharged patients at a hospital with basic and standard care. Specially trained nursing personnel who diagnosed hyperactive delirium using the confusion assessment method (CAM) by Inouye [9, 10, 13] worked at the hospital. If three or more of the four questions of the CAM questionnaire were answered positively in anomalous patients, the patient was considered delirious. Trained personnel not only documented the occurrence of delirium but also the time-related expenses during treatment. A matrix with 10 areas representing time units that was developed together with the nursing staff was used at the hospital: (1) Observation/monitoring, (2) documentation, (3) communication (e.g. physicians, next of kin and telephone conversations), (4) medical measures, (5) mobilization/assistance, (6) safety measures (e.g. fixation, increased presence, transfer to an intensive care ward or a single bed room), (7) administration of medications, (8) personal care (e.g. reassurance and guidance), (9) increased basic care and (10) two staff members needed for care. This documentation of time spent was performed by the nursing staff as well as the physicians from the beginning to the end of the episode of delirium. Thus, if a patient had several episodes of delirium, each episode was collated and documented individually. During a daily transfer from the nursing team to the physician team, new delirium patients were discussed and colleagues were informed of details regarding patient status and the course of the treatment. Positive results of screening were medically reviewed and approved.

The available data on main diagnosis, age, gender, comorbidity, case mix, patient comorbidity and complexity level (PCCL) were used to group the patients in the German diagnosis-related groups (G-DRG) system. Different PCCL groups were automatically formed (0–4), with “4” indicating the most complex and comorbid cases on one side of the matrix and the highest cost on the other side of the matrix. In this manner all the retrospectively analyzed patients could be allocated to returns, defined as case mix points. Prevalent and incidental delirium were differentiated in the retrospective analysis: prevalent delirium was defined as delirium existing on admission and incidental delirium was defined as new onset delirium occurring during hospitalization. These terms were implemented to retrospectively differentiate between patients with delirium at admission and those with delirium diagnosed during the follow-up of clinical treatment with respect to costs.

## Results

Data for patients discharged over the course of 3 months were collected in the retrospective database analysis (*n* = 568). The average age of the patients was 69.3 years, 290 patients were women and 278 patients were men. There were 82 delirium patients (37 women and 45 men) in the database where 20 of these delirium cases were detected on hospitalization (prevalent delirium) and 62 were detected during hospitalization (incidental delirium).

## Duration of delirium and time of onset

**Fig. 1:**
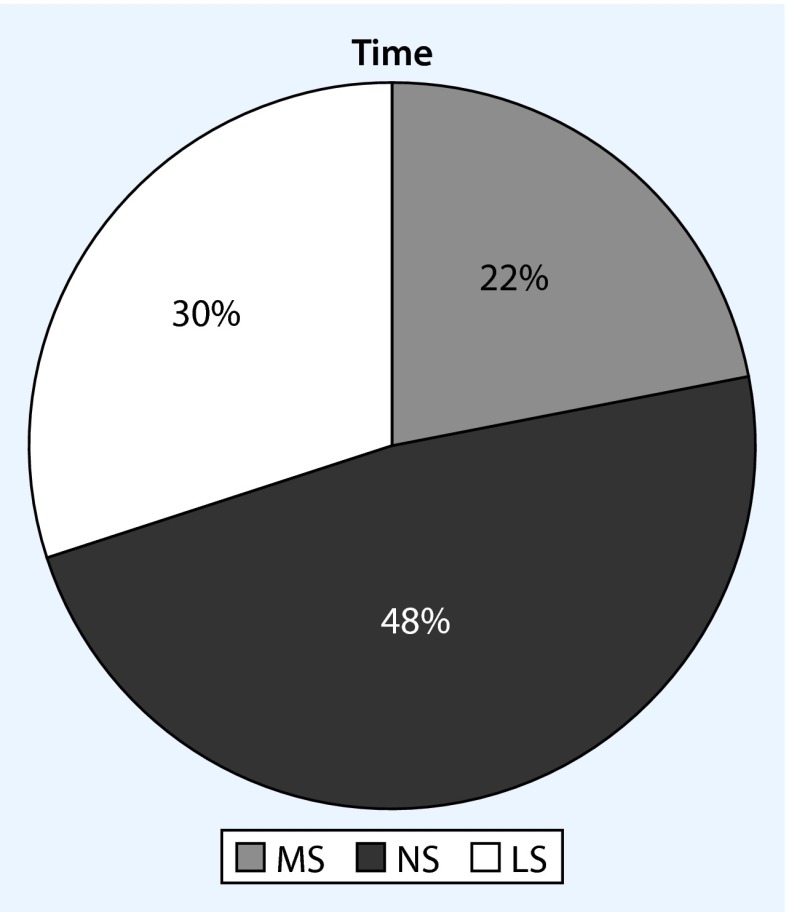
Incidence of delirium episodes depending on shift (*MS* morning shift, *LS* late shift, *NS* night shift)

A total of 191 cases of hyperactive delirium during treatment were documented; thus, each delirium patient had an average of 2.27 hyperactive delirium episodes. The analysis showed that patients with delirium on hospitalization had a significantly lower average number of delirium episodes (*n* = 1.75) than patients for whom delirium was only detected after hospitalization (*n* = 2.31). This difference was significant (*p* < 0.001). According to the collected data, the average duration of delirium episodes was almost identical regardless of the time of onset: 1.65 days ± 1 day. There was no significant difference in delirium duration between cases of prevalent and incidental deliria. The analysis of the correlation between the number of episodes of delirium and the individual work shifts showed that most episodes occurred during the night shift, followed by the late and morning shifts (Fig. 1). These differences were not significant.

**Fig. 2:**
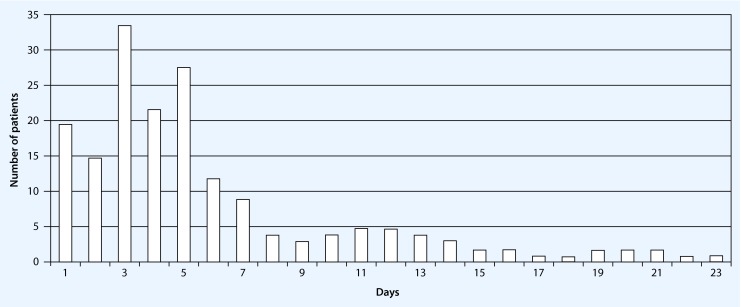
Time window (days) until onset of hyperactive delirium after hospitalization

There was an increase in incidental delirium in the first 5 days after admission and delirium occurred on average 4.15 days after hospitalization at the clinic. For all documented delirium episodes 76.9 % occurred within the first week and 15.38 % occurred within the second week (Fig. 2).

## Age and duration of hospitalization of delirium patients

The average age of all patients studied was 69.3 years. The hyperactive delirium patients were on average 78.8 years old and, therefore, significantly older than the overall patient population by almost 10 years (*p* < 0.01). The average duration of hospitalization (DH) for non-delirium patients was 8.5 days and the DH was distributed according to age: 69-year-old non-delirium patients had a DH of 8.0 days and 77-year-old (range 75–84 years old) non-delirium patients had an average DH of 10.4 days. These data are the annual average data of the different age groups in the ward. Delirium patients with the same average age (77 years) had an average DH of 12.7 days. Thus, there was an age-correlated, significant average extension in the DH of 2.3 days in the delirium patients compared to the non-delirium patients (*p* < 0.01) for patients who were an average of 77 years of age. In comparison to normal patients, the DH of all delirium patients was on average 4.3 days longer than that of normal patients (12.7 versus 8.5 days, respectively). The cases of prevalent and incidental delirium also differed significantly with regards to DH: 6.85 versus 13.61 days, respectively (*p* = 0.002). The average case severity or case mix index (CMI) was significantly higher in delirium patients than in normal patients (1.35 versus 0.83, respectively).

## Typical main diagnoses

The following main diagnosis groups according to the international classification of diseases (ICD-10) [7] and the G-DRG [8] were most common in hyperactive delirium patients:

1. Stroke (transitory ischemic attack/apoplexy)
2. Heart insufficiency (decompensated heart insufficiency/non-ST segment elevation infarction)
3. Pulmonary diseases (pneumonia/chronic obstructive pulmonary disease).

Patients with hyperactive delirium in combination with stroke, heart insufficiency and pneumonia had an extended DH of + 2.1 days (*p* = 0.1), + 2.1 days (*p* = 0.163) and + 3.9 days (*p* = 0.105), respectively, in comparison to non-delirium patients; however, these differences were not significant. Considering the additional diagnoses in the two groups, there were on average 12.7 additional diagnoses and a PCCL of 2.94 in patients with prevalent hyperactive delirium. In contrast, patients with incidental hyperactive delirium had 10.52 additional diagnoses and a PCCL value of 3.33.

## Material costs

The cost hierarchical analysis of drug use showed that antibiotics (intravenous) had the highest cost, followed by intravenous fluid (isotonic, physiological) and drug administration (neuroleptics, benzodiazepines). Because there were significant differences between cases of prevalent and incident delirium (1.75 versus 2.31 episodes of delirium, respectively) the costs were also calculated for each delirium episode. Prevalent delirium required fewer expensive medications and antibiotics. The average cost accrued for all drugs administered was 1986.67 €. On average, the cost of drugs administered to cases of prevalent delirium was 10.94 € and that for incidental delirium was 28.51 €. Considering the significant difference in the numbers of prevalent and incidental delirium events, the costs per delirium episode were 18.28 € for prevalent delirium and 24.10 € for incidental delirium (Table 1).

**Table 1:** Drug costs in prevalent and incidental delirium

	Number of delirium events	Therapeutic costs total (1986.67€/190 events) 10.45€/delirium	Therapeutic costs/case	Share of antibiotics
Prevalent delirium patients (*average number of events)	20 (*1.75) = 35	35×10.45 € = 365.75 €	365.75 €/20 = 18.28 €	0 %
Incidental delirium patients (*average number of events)	62 (*2.31) = 143	143×20.45 € = 1494.35 €	1494.35 €/62 = 24.10 €	79 %

To receive a possible business case (expenditure/year) concerning medication costs for hypermotoric delirium the costs were predicted on the basis of the calculated data. A projection of the expected total number of delirium cases in this age group of approximately 2300 patients/year (presumed average value one third delirium incidence, the incidence/prevalence significantly depends on average age and the previous illness, previous treatment and can fluctuate between 17 % and 80 % according to the literature) resulted in approximately 760 delirium patients/year or approximately 190 delirium patients/quarter year. Thus, subtracting the 82 documented patients approximately 108 further delirium patients could be expected. If the number of 760 patients is multiplied with a presumed mean average cost of 23.72 € (number of delirium events/patient×mean cost/delirium = 2.27 €×10.45 € = 23.72 € the additional annual medication costs for all cases of expected hyperactive delirium are approximately 18,000 € for a ward of this size and patient distribution calculated from the available data.

## Personnel time expenditure

**Fig. 3:**
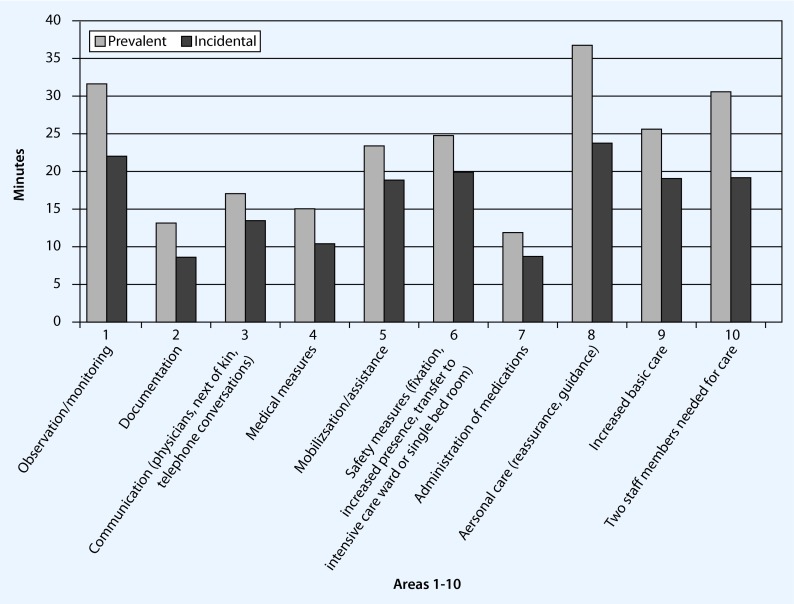
Time expenditure per prevalent/incidental delirium episode in 10 areas

A patient with hyperactive delirium required on average approximately an additional 240 min of documented, additional care and/or treatment by the nursing staff. Prevalent delirium patients required approximately 260 min, incidental delirium patients on the other hand approximately 215 min of additional personnel expense per case (Fig. 3). Patients with prevalent delirium required consistently and significantly more time contingents than patients with incidental delirium and the time expenditure in all documented areas was on average constantly 38.12 ± 9.7 % higher.

The greatest time requirement was in the three areas of care, monitoring/observation and commitment of two staff members (Table 2). The differences were significant.

**Table 2:** Significant differences between prevalent and incidental delirium in selected areas

Delirium type	Care (min)	Monitoring/observation (min)	Commitment of two staff members (min)	Safety measures (min)	Documentation (min)
Prevalent delirium	66.67	45.71	92.50	39.00	19.00
Incidental delirium	22.94	20.45	18.59	18.65	8.44
*p*-value	**0.012**	**0.048**	**0.045**	**0.049**	**0.004**

The projection of the average time expenditure for all delirium patients (*n* = 760) gave a total of approximately 760 × 240 min = 182,400 min/year. This corresponds to 3040 h/year. With a total working time of 1617 h/year (38.5 h/week and 30 days of vacation resulting in a total working time of 1848–231 h) this means an average time expenditure of 1.8 positions of a registered nurse. The calculated amount is 81,000 € with a presumed gross annual total of 45,000 €. Concerning the additional wage costs the total amount is 96,000 € and results in nursing personnel costs of 96,000 €/760 = 126.3 € per delirium case.

The calculated average time value per delirium patient was 66.40 min for physicians. There were no differences between the delirium types (incidental/prevalent) for the physician time expenditure. This resulted in a calculated total time expenditure of 50,454 min or 841 h for 760 cases of delirium. This corresponds to almost exactly 0.5 physician positions/year. With a presumed gross income of 80,000 € and concerning the additional wage costs the total amount is 48,000 € resulting in average physician costs of 65.15 € per delirium case when divided by 760.

## Total costs

Without calculating the effects of the length of stay, the total costs for delirium are composed of the following: 60 % nursing personnel costs, 30 % medical services and 10 % additional medication. The average costs per patient in German hospitals were calculated [5]. The average additional costs per delirium patient are thus approximately 194 €. The daily costs were calculated at an average of 260 €. This value arises from the calculation of the average costs of a normal DRG case on this ward (1.1 casemix points CMP with a DH of 8.5 days corresponding to 3430 €/8.5 = 400 €) and the average, internal clinical day/boarding costs (approximately 120 €/day) and resulted in the average data together with the extension in the duration of hospitalization of the delirium patients given in Table 3.

**Table 3:** Costs (personnel + drugs + duration of hospitalization) of trained personnel

	Costs/prevalent delirium patient (*n* = 20)	Costs/incidental delirium patient (*n* = 62)	Average values
Care (32 €/h)	128 € (64 %) 260 min or 4 h)	112 € (54 %) (215 min or 3.5 h)	Approx. 60 %
Physicians (43 €/h)	56 € (30 %) (66 min or 1 h)	56v€ (30 %) (66 min or 1 h)	Approx. 30 %
Medication	18.28 € (6 %)	24.10 € (16 %)	Approx. 10 %
Total	**202.28 €**	**192.10 €**	**Approx. 194 €**
DH extension	**–**	**1118 €** **+ 4.3 days**	
Individual case	**Approx. 180 €**	**Approx. 1300 €**	**Approx. 1000 €/delirium patient**
Total cost			**Approx. 1200 €/delirium patient**

If one considers the main diagnosis (i.e. stroke group, heart insufficiency group and lung group) in addition to the delirium type (prevalent vs. incidental) the influence of the point in time when delirium was diagnosed can be seen. Patients with prevalent delirium are hospitalized for less time and those with incidental delirium for a longer time than the average patient (Table 4).

**Table 4:** Duration of hospitalization and cost differences between prevalent and incidental deliria in stroke, pneumonia and heart insufficiency

Variation of the length of stay in the German diagnosis-related groups main diagnosis						
Main diagnosis	Stroke	Stroke	Pneumonia	Pneumonia	Heart insufficiency	Heart insufficiency
Delirium type	Prevalent	Incidental	Prevalent	Incidental	Prevalent	Incidental
Duration of hospitalization	––	+ 2.13 days	− 4 days	+ 3.93 days	− 4 days	+ 2.12 days
Costs		+ 550 €		+ 1000 €		+ 550 €

If this result (1200 €/delirium patient) is projected out for 1 year (790 patients with delirium), the result is a loss of 948,000 €.

## Statistical considerations

The present work examines the financial side of hyperactive delirium on hospitalization and during the clinical course. Specifically, personnel expenditure, material costs and the duration of hospitalization in hyperactive delirium were collected. Patients without delirium and with delirium were compared and the following characteristics were examined:

1. Time of onset of deliria: night, morning and late shift.
2. Influence of age factor on the presence of delirium
3. Influence of delirium on duration of hospitalization
4. Additional time expenditure by personnel

The statistical analysis was performed by Murat Karaman (M. Sc. statistics) Berlin. The statistical analysis was performed by means of the Mann-Whitney test and the χ^2^-test. The SPSS 15.0 was used for the analysis of data.

## Discussion

What does a delirium patient cost? The retrospective data analysis shows that hyperactive delirium patients are expensive. According to the available data an additional cost of approximately 1200 € per diagnosed delirium patient must be taken into account. The main cost factors are personnel expenditure and the DH. Altogether, the additional costs for a general internal ward with an average occupancy of 80 % per year are approximately 948,000 €. This number depends on the patient clientele: it is probably higher in neurological patients and postoperative patients but lower in conservatively treated geriatric patients. The differentiation between prevalent and incidental deliria in this work provides useful aspects in the evaluation of deliria. The personnel expenditure is higher in patients who have prevalent delirium on hospitalization but the DH and total costs are lower. Incidental delirium that develops during treatment requires much longer hospitalization and is thus more expensive. The following results are to be discussed:

1. The first days are decisive in the onset of delirium.
2. Early diagnosed delirium is managed differently: maybe there is a higher awareness of delirium diagnosed on admission than of delirium diagnosed on the ward in the night shift. Perhaps although physicians take the delirium on admission more seriously, these presumptions may have a real background in the routine clinical work. The effect of the significantly earlier discharge of patients with delirium detected early alone suggests that the costs would drastically rise when untrained personnel are used and if the delirium is not detected.

Because only hypermotoric delirium was diagnosed the total amount of expected delirium numbers was relatively low and only 14 % of the patients exhibited delirium. According to clinical experience hypomotoric patients are in a worse functional condition, are hospitalized even longer and have even more complications; however, because they are more difficult to diagnose, collecting information on cost-saving for these patients requires much more experience and additional trained personnel. There is evidence from clinical experience and other studies that the proportion of hypomotoric delirium is probably higher than that of hypermotoric delirium and may be even more expensive. This limits the significance of the data analysis to hypermotoric delirium and indicates that the total costs for delirium patients may be much higher than calculated here. Therefore, a similar data analysis for hypoactive delirium would be important in the next step.

Delirium represents a typical problem in older patients [3, 10, 17]. Only the data of patients who had already been discharged could be collected because medical personnel were professionally trained to diagnose delirium and to document this. The CAM score was used at this clinic, whereby subsymptoms could also suggest the presence of delirium. New examinations by Han et al. [4] support this finding that each patient with vigilance disorders should be monitored. This retrospective database analysis emphasizes the importance and effectiveness of training personnel in the clinical treatment of patients with delirium.
